# Assessing the Impact of Nutritional Support Teams on Clinical Outcomes: Compliance and Feasibility of Micronutrient Supplementation

**DOI:** 10.3390/jcm13123422

**Published:** 2024-06-11

**Authors:** Sunmin Lee, Jongbeom Shin, Mina Kim, Suejin Jo, Soo-Hyun Park

**Affiliations:** 1 College of Pharmacy and Research, Institute of Life and Pharmaceutical Sciences, Sunchon National University, Suncheon 57922, Republic of Korea; 2 Division of Gastroenterology, Department of Internal Medicine, InHa Hospital, Incheon 22332, Republic of Korea; 3 Department of Nursing, InHa Hospital, Incheon 22332, Republic of Korea; 4 Clinical Nutrition Department, Dongduk Women’s University Graduate School, Seoul 02748, Republic of Korea; 5 Department of Neurology, Soon Chun Hyang University Hospital, Seoul 04401, Republic of Korea

**Keywords:** nutritional support team, micronutrient, malnutrition, multivitamins, trace elements, intensive care unit, clinical outcomes

## Abstract

**Background**: Micronutrient (MN) supplementation has a positive impact on clinical outcomes. However, the evidence for the impact of MN supplementation remains controversial. Therefore, our study aims to assess the impact on nutritional outcomes according to exploring the implementation of MN support with multidisciplinary collaboration. **Methods**: This retrospective cohort study was conducted at a university hospital in Incheon, Korea. All patients referred to a nutrition support team (NST) between July and November 2022 were included. The NST reviews the MN protocol, which includes multivitamins and trace elements, based on international nutrient guidelines. All patients who were on nothing per oral and did not meet ≥70% of their nutritional requirements within 1 week were recommended MN supplements. Compliance with the MN protocol was evaluated, alterations in nutritional status based on the Nutrition Risk Screening 2002 (NRS 2002) scoring system and clinical outcomes were assessed after 7 day and at discharge. Multiple logistic regression analysis was used to identify factors associated with high nutritional risk in discharged patients. In addition, a sub-analysis was performed on changes in the nutritional of patients on the ward and in the ICU. **Results**: A total of 255 patients were eligible for analysis, with many patients requiring an MN supply of nothing per oral. The rate of implementation of MN supplementation was 50.2%. The findings indicate a significant decrease in the NRS 2002 score in the good compliance group with MN supplementation. No significant differences in protocol compliance were observed in terms of mortality, hospital stay, or length of stay in the intensive care unit. However, bad compliance with MN supplementation was correlated with risk factors for malnutrition at discharge. In subgroup analysis, nutritional status in the ICU and wards improved, with a significant difference between the two groups. **Conclusions**: The implementation of a MN supplementation protocol by a multidisciplinary NST is a feasible approach for improving the nutritional status of inpatients. Ensuring high compliance with this protocol is crucial, as poor compliance has been identified as a risk factor for malnutrition at discharge. Active intervention by the NST is essential to achieve optimal nutritional outcomes.

## 1. Introduction

Nutritional insufficiency is related to specific diseases, including anemia, obesity following bariatric surgery, chronic liver disease, kidney disease, inflammatory bowel disease, cardiomyopathies, and heart failure. Failure to meet nutritional requirements can contribute to the aggravation of diseases among inpatients [1]. Moreover, proper nutritional supplementation has shown favorable clinical outcomes, including morbidity, mortality, and readmission rates, in the intensive care unit (ICU) [2,3,4,5]. Most nutritional supplements tend to target patients’ total calorie and protein nutritional needs [3].

Malnutrition often correlates with deficiencies in both micronutrients and macronutrients [6]. Nutrient support guidelines recommend micronutrient (MN) supplementation, particularly in patients with long-term malnutrition [7,8,9]. Guidelines recommend MN supplementation for malnourished patients with daily requirements for intravenous nutrition and low caloric intake [9]. A consensus on the daily recommended intake has also been established to guide the supply of MNs.

Despite the positive clinical outcomes observed with nutritional supplements, MN supplementation remains controversial owing to statistically inconsistent clinical outcomes [10]. There is insufficient evidence regarding the clinical benefits of high-dose supplementation of trace elements [11]. No clinical benefit was found for additional micronutrient supplementation. However, previous studies have not included specific protocols based on guidelines for micronutrients, and further research based on consistent criteria is needed [6].

We presented a protocol for the provision of MN based on international guidelines in collaboration with a multidisciplinary nutrition support team (NST). The objective of this study is to assess the feasibility and effectiveness of nutritional interventions implemented by a multidisciplinary NST. The first objective is to determine the compliance rate of patients with the MN supplementation protocol. The second objective is to assess the impact of MN supplementation on changes in nutritional status, as measured by the Nutrition Risk Screening 2002 (NRS 2002) scoring system. Thirdly, clinical outcomes following MN supplementation are assessed, including mortality rates, hospital stay duration, and length of stay in the intensive care unit (ICU). Lastly, the aim is to identify factors associated with high nutritional risk at discharge among patients receiving MN supplementation. This analysis will enable a better understanding of which patients are most at risk and may benefit the most from targeted nutritional interventions.

## 2. Materials and Methods

### 2.1. Study Design and Participation

All patients referred to the NST between July and November 2022 were included. Among malnourished patients referred to the NST, all patients (1) who received MN-free parenteral nutrition and (2) who did not meet 70% of their nutritional requirements (energy and protein) via enteral nutrition were classified as individuals eligible for MN supplementation [7]. Patients with incomplete information in their electronic medical records, as well as pediatric patients, were excluded. The implementation of the prescribed supplementation within 7 days of the NST recommendation was checked. Patients who successfully received the prescribed supplementation were categorized into the good compliance group, whereas those who did not receive supplementation were classified into the bad compliance group. This study is to evaluate the compliance of the multidisciplinary interventions and to identify changes in nutritional status and factors associated with nutritional risk groups after compliance with the intervention protocol. In addition, we also analyze the difference in change in nutritional status between patients in ICU and wards.

### 2.2. Nutrient Supplement Protocol

To develop a nutrient support protocol that includes MNs according to international guidelines (ASPEN, American Society for Parenteral and Enteral Nutrition; ESPEN, European Society for Parenteral and Enteral Nutrition) [8,9], a multidisciplinary protocol council comprising physicians, pharmacists, nurses, and nutritionists was established. In our team, physicians, nurses, and nutritionists conducted nutritional assessments, administered nutritional support, and monitored patient progress, ensuring that the interventions were tailored to the clinical situation. Pharmacists were actively involved in the selection of nutritional supplements and medications. Additionally, the development and discussion of all nutritional support protocols involved input from all professional disciplines. Caloric and protein requirements were expressed as percentages to ensure adequacy. In cases where patients had a body mass index (BMI) > 25, the adjusted weight was used. Caloric intake ranged from 25 to 30 kcal per kilogram of body weight, while protein requirements were determined to be between 0.8 and 1.2 g per kilogram of body weight, considering renal function [7,12].

Among the various multivitamin products available in Korea, daily supplementation with products that fulfill the daily requirements of adults was recommended [9] (Table S1). However, for trace elements, it was challenging to identify a suitable product that could adequately meet the required dosage among the available options within a hospital setting. Consequently, a weekly supply of trace elements was adopted as a general practice [13]. Selenium was selected as the standard product for supplementation due to the lack of a combination product. Selenium, as described in Table S1, was selected as the standard product in Korea that met the recommended daily allowance for supplementation.

We recommended the parenteral route for PN, while allowing any form of MN to be administered according to clinical practice. Monitoring and verifying trace element levels after supplementation is recommended, particularly in situations anticipating significant deficiency, like continuous renal replacement therapy, burns, gastrointestinal surgery, and excessive gastrointestinal loss [14]. After 1 week, the implementation of all recommendations was assessed, and a nutritional evaluation was repeated at the end of the study in instances of discharge or mortality.

## 3. Data Collection

Baseline characteristics, such as age, sex, weight, and BMI, were documented. To assess the clinical condition, the Charlson Comorbidity Index (CCI), Acute Physiology and Chronic Health Evaluation III (APACHE III) score, and use of a mechanical ventilator were evaluated in patients admitted to the ICU. For nutritional status assessment, data on feeding status and laboratory findings, such as albumin and total protein levels, were collected. Nutritional status was assessed based on the Nutrition Risk Screening 2002 (NRS 2002) scoring system [15].

## 4. Statistical Analysis

For descriptive statistical analysis, continuous variables were expressed as mean (standard deviation) for normally distributed variables and median (interquartile range) for non-normally distributed variables, while categorical values were presented as percentages. To compare groups, the chi-square, and Fisher’s exact tests were employed for categorical variables, while the *t*-test was used for continuous variables. Paired *t*-tests and chi-square tests were used to compare the mortality rate, NRS 2002, duration of hospitalization, and length of ICU admission. Kaplan–Meier curves were used to investigate the relationship between overall survival and the administration of MN supplements. The impact of nutritional interventions on ICU and ward patients, excluding those who had expired, was assessed using repeated measures ANOVA for the analysis of NRS. Multivariate logistic regression analyses were performed to identify the risk of malnutrition in the NRS 2002 evaluation. Variable selection was based on items with a *p*-value < 0.1 in the binary regression analysis. Two-sided *p*-values were used to determine statistical significance. All statistical analyses were conducted using IBM SPSS Statistics V21.0 software.

## 5. Results

**Figure 1:**
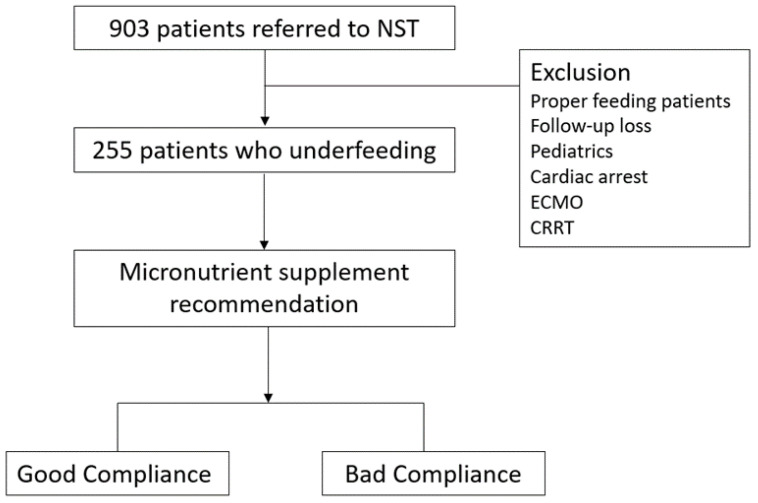
Flow of micronutrient supplement plan. NST, nutritional support team; ECMO, extracorporeal membrane oxygenation; CRRT, continuous renal replacement therapy.

During the study period, 903 malnourished patients were referred to the NST. Among these, 63 were excluded from the analysis based on the exclusion criteria (Figure 1). In this study, 255 patients required MN supplementation, with 148 in the ward and 107 in the ICU. Of these, 80 patients (31.4%) receiving enteral nutrition (EN) did not meet the nutritional requirements for over 1 week, whereas 162 patients (63.5%) received MN-free parenteral nutrition (PN). The EN group had a lower rate of nutritional adequacy compared to the PN group. Sex differences were observed between the good and bad compliance groups to meet the NST recommendations, although no significant differences were noted in other variables. Further details are listed in Table 1.

The NRS score at reference showed no significant difference between the two groups (*p* = 0.605). There were no significant differences in the duration of hospitalization and ICU stay between the two groups. However, there was a difference in NRS at discharge and in the change in score depending on compliance, as shown in Table 2.

**Figure 2:**
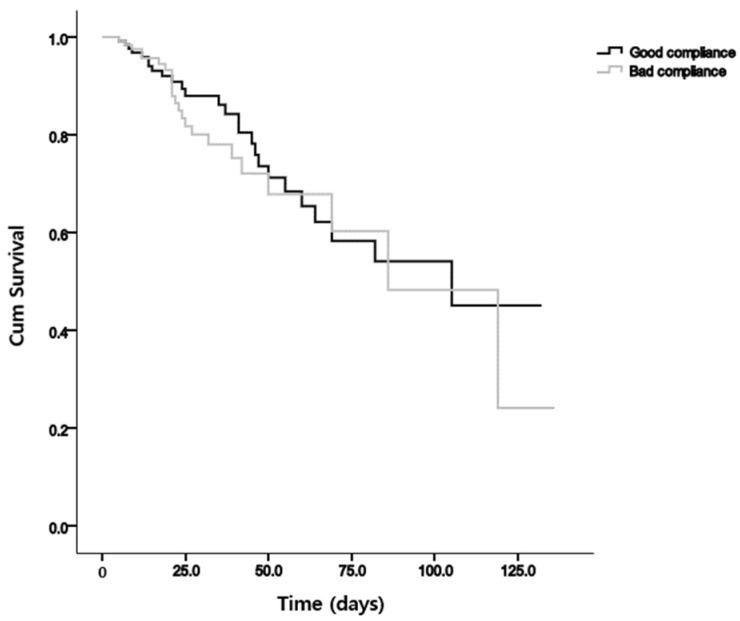
Plots of Kaplan–Meier survival between good compliance and bad compliance group (*p* = 0.536).

**Figure 3:**
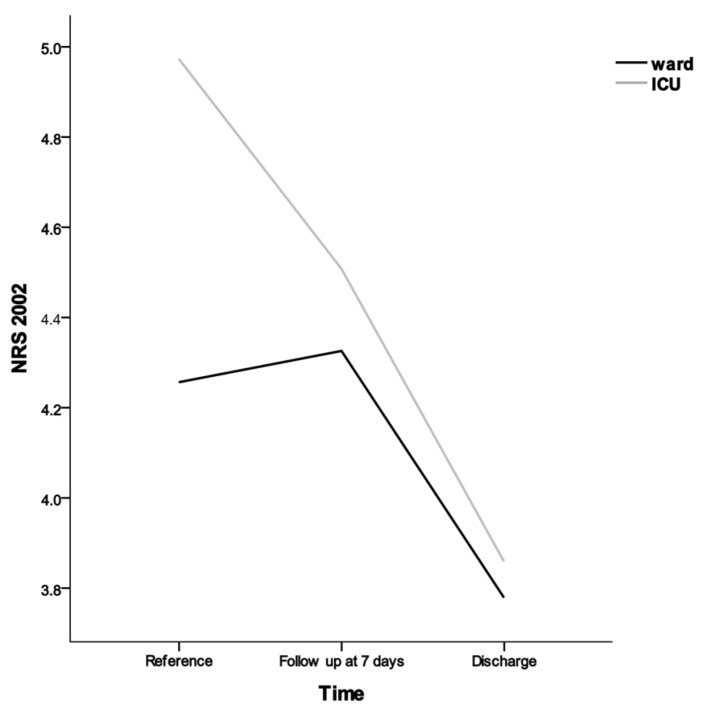
Differences in the effectiveness of nutritional interventions between ICU and ward. NRS 2002, Nutrition Risk Screening 2002; ICU, the intensive care unit. ICU patients (*n* = 71), ward patients (*n* = 86).

Regarding mortality rates, no significant difference was observed between the good compliance and bad compliance groups. The Kaplan–Meier curves of the overall survival according to interventions are shown in Figure 2. No statistically significant difference in overall survival was observed between the bad compliance group and the good compliance group (*p* = 0.536). However, significant differences over time were observed in the influence of ICU and ward patients, as evidenced by notable distinctions in NRS assessments (Figure 3).

The results of identifying the nutritional factors associated with the high-risk group based on NRS 2002, as assessed during discharge, are presented in Table 3. Both the group with bad compliance and the presence of nutritional risk on hospitalization were identified as risk factors for being classified in the high-risk group even at discharge, with respective odds ratios of 1.54 (95% confidence interval [CI] 1.74–12.63, *p* = 0.002) and 1.55 (95% CI 1.05–2.29, *p* = 0.024).

Conversely, a higher BMI at admission was found to be a variable associated with a lower risk of being categorized in the high-risk group (odds ratio 0.83, 95% confidence interval 0.73–0.93, *p* = 0.003).

## 6. Discussion

This study demonstrates that MN supplementation under the guidance of a multidisciplinary council is correlated with improving factors for malnutrition at discharge according to compliance. In particular, nutritional status in the ICU and wards improved significantly, as shown in the NRS 2002 scores change through NST. This study focused on the continuous monitoring of nutritional and clinical outcomes.

The nutrition intervention protocol includes a plan for providing MN to malnourished patients. MNs play a crucial role in nutrient metabolism and function, and according to guidelines, their supplementation is recommended for hospitalized patients who are at risk of deficiency [7,8,9]. While the clinical benefits of additional supplementation or high doses of single antioxidant nutrients remain inconclusive, guidelines suggest meeting the daily requirements to prevent malnutrition without adverse effects [11]. In this study, patients receiving MN-free PN or EN, who were expected to experience long-term nutritional deficiencies, were selected as participants for investigation [16,17].

Multidisciplinary efforts have been made to address the nutritional needs of these patients. In addition to identifying high-risk patient groups, multi-vitamin products that fulfilled the daily requirements were carefully chosen. Concerning trace elements, products containing manganese, chromium, zinc, and copper were administered once a week to supplement the recommended dosage without anticipated toxicity while closely monitoring liver and renal function indicators [13,14]. Furthermore, selenium supplementation is recommended as part of the recommended supplemental regimen. Based on individual assessments, vitamin K was advised to provide the minimum required amount [7]. Achieving an optimal vitamin supply proved challenging, especially when dealing with trace elements affecting the kidneys and liver.

The guideline recommended that micronutrients should be supplied in the recommended daily allowance and provided periodically, even in situations of product shortage [8,13]. Our protocol recommended staying within the range of daily requirements for trace elements (Table S1). Numerous discussions are necessary to identify suitable products for supplementation, considering factors such as reasonable pricing and determining the most effective products for clinical application. Through multidisciplinary collaboration, we aimed to generate exemplary instances that facilitated the drawing of meaningful conclusions.

A monitoring plan was developed through multidisciplinary collaboration, including follow-up 7 days after referral and at discharge. Nutritional assessments were conducted using the NRS 2002 after nutritional interventions. Clinical outcomes, such as mortality, length of stay, and ICU stay, were monitored. Finally, significant differences were found in the NRS 2002 scores change, assessing a positive nutritional outcome in our study by monitoring the food intake status over the past week.

Of all the referred patients, 30.4% (255 out of 840) were found to require MN, indicating the presence of anticipated malnutrition. Among these, a significant percentage (63.5%, 162 out of 255) had PN. Active intervention is crucial for MN supplementation, as it is necessary to provide the required amount when PN is administered without MN [8,18]. The compliance rate of the recommendation was 50.2% (128 out of 255 cases), and no significant differences were observed among the patient groups, except for variations in the proportion of sex.

The NRS 2002 is a widely recommended tool for assessing and predicting malnutrition in hospital settings [19]. It has also been extensively examined as a means of predicting long-term clinical outcomes [20]. Within the context of this study, a significant reduction in the scores of the good compliance group was observed following the intervention, indicating a positive impact. During the intervention period, there were consistent nutritional impacts, especially among ICU patients compared to ward patients (Figure 3). Furthermore, patients with a shorter hospitalization period or longer ICU admission period demonstrated a lower acceptance rate of NST and required active intervention.

Previous studies have indicated a positive correlation between NST activity and clinical outcomes, with acceptance of the protocol suggested as a crucial factor in this relationship [3]. In the present study, a higher mortality rate was observed in the good compliance group than in the bad compliance group, although the difference was not statistically significant. Nevertheless, significant observations were made regarding the conduct of physicians in accepting interventions, and these findings should be acknowledged in future endeavors targeting at-risk groups where interventions have not been carried out. These findings highlight the importance of implementing diverse strategies to enhance patient acceptance and adherence to the protocol in future interventions.

The nutritional status of the hospitalized patients was closely monitored by assessing their NRS 2002 scores at the time of referral to the NST and after the observation period. Among these patients, those identified as high-risk (with a score of ≥ 5) were selected for further analysis because previous studies have associated this high-risk group with adverse clinical outcomes, including mortality [20,21,22,23]. Despite the absence of a significant difference in the referral NRS 2002 scores between the two groups, bad compliance emerged as a risk factor for the high-risk group after accounting for the other significant variables. These findings are consistent with those of previous studies that focused on the high-risk group identified by the NRS 2002, demonstrating a propensity for reduced malnutrition risk following intervention [2].

This descriptive study examined the nutritional clinical status and clinical outcomes following interventions by a multidisciplinary team with a particular focus on trace element supplementation based on guidelines. However, this study has certain limitations. First, the NRS 2002 is usually used as an index to screen for malnutrition, assessing the overall nutritional status in the acute phase individual [24,25]. It compromised nutritional impairment, severity of illness, and nutritional status for one week. Although the NRS2002 has been evaluated as a predictor of clinical outcomes [26,27], it cannot differentiate between clinical and nutritional intervention results [28]. Further research is needed to determine whether this improvement is the result of the nutrition team’s intervention efforts. Second, the follow-up of MN supplementation was limited to 7 days based on NRS 2002, highlighting the need for continuous monitoring in patients with long-term hospitalization. Individual monitoring of suspected deficient trace elements was not performed despite recommendations. Therefore, further research in the form of prospective intervention studies is required to address this issue based on the most recent MN supplement guideline [29]. In addition, although we accounted for the clinical condition of patients using the CCI and APACHE scores, patients could be transferred between the ICU and the ward during hospitalization. This reclassification at the time of referral resulted in a heterogeneous patient population, which we acknowledge as a limitation of our study. Given the retrospective observational nature of this study, further validation through randomized intervention studies is warranted to strengthen our findings. The results emphasize the crucial role of a multidisciplinary team in recognizing malnourished patients at the time of referral and enhancing the acceptance rate of NST activities. In conclusion, we confirmed the feasibility of the MN supplementation protocol through multidisciplinary collaboration. Also, compliance with MN supplementation is associated with the risk factor of malnutrition at discharge. Therefore, MN supply protocol and compliance is need to improve malnutrition for hospital patients.

## Figures and Tables

**Table 1:** Baseline characteristics.

Value	Total (*n* = 255)	Good Compliance (*n* = 128)	Bad Compliance (*n* = 127)	*p*-Value
Age, year	70.1 ± 15.5	68.5 ± 16.2	71.8 ± 14.6	0.087
Male, *n* (%)	150 (58.8)	67 (52.3)	83 (65.4)	0.047
Weight, kg	58.3 ± 27.8	60.2 ± 36.4	56.5 ± 14.7	0.296
BMI, kg/m^2^	21.7 ± 6.5	21.9 ± 6.0	21.6 ± 7.0	0.692
Feeding status				
Enteral, *n* (%)	80 (31.4)	33 (25.8)	47 (37.0)	0.29
Non per oral, *n* (%)	162 (63.5)	88 (68.8)	74 (58.3)	
Others, *n* (%)	13 (5.2)	7 (5.4)	6 (4.7)	
Enteral achievement rate,				
Energy, kcal/kg	43.0 (26.0–58.0)	42.0 (29.0–51.0)	46.0 (26.0–60.5)	0.417
Protein, g/dL	44.0 (25.0–60.0)	46.0 (25.0–58.0)	44.0 (25.0–60.5)	0.926
Parenteral achievement rate				
Energy, kcal/kg	55.0 (40.0–73.0)	58.0 (40.5–74.0)	51.0 (40.0–71.0)	0.528
Protein, g/dL	62.5 (43.0–84.0)	62.0 (43.0–84.0)	63.0 (44.0–83.0)	0.77
CCI	5.0 (4.0–7.0)	5.0 (4.0–7.0)	5.0 (4.0–7.0)	0.971
APACHE II	19.0 (14.0–24.0)	19.0 (13.5–25.5)	19.0 (14.0–24.0)	1
NRS 2002	5.0 (4.0–5.0)	4.5 (4.0– 5.0)	5.0 (4.0–6.0)	0.111
Ventilator care, *n* (%)	63 (25.7)	26 (21.1)	37 (30.3)	0.279
Total Protein, g/dL	6.4 (5.7–7.0)	6.5 (5.7–7.0)	6.3 (5.8–7.0)	0.556
Albumin, g/dL	3.3 (2.6–3.8)	3.4 (2.6–3.9)	3.2 (2.6–3.8)	0.459

Values are presented as mean ± standard deviation, number (%), or median (interquartile range). APACHE II score, Acute Physiology and Chronic Health Evaluation II score; BMI, body mass index; NRS, nutritional risk screening; CCI, Charlson Comorbidity Index.

**Table 2:** Clinical outcomes after intervention of nutrition support team focusing on micronutrients supplements ^a^.

Value	Total (*n* = 204)	Good Compliance (*n* = 100)	Bad Compliance (*n* = 104)	*p*-Value
NRS 2002 at referral	4.5 ± 1.3	4.5 ±1.1	4.6 ± 1.5	0.605
NRS 2002 at discharge	3.8 ± 1.2	3.5 ± 1.2	4.1 ± 1.2	0.002
NRS 2002 change	−0.7 (1.5)	−0.7 (1.5)	−0.4 ± 1.5	0.001
Difference between input and output at referral, mL		533.5 ± 105.6	272.9 ± 77.4	0.048
Difference between input and output at discharge, mL		277.8 ± 86.1	276.1 ± 84.4	0.954
Length of hospital stay, day	22.0 (14.0–41.5)	23.0 (15.0–47.0)	21.0 (13.0–36.0)	0.137
Length of ICU stay, day	5.0 (0.0–15.0)	4.0 (0.0–15.0)	6.0 (0.0–15.0)	0.384
Mortality ^b^, *n* (%)	51 (20.0)	28 (21.8)	23 (18.1)	0.552

Values are presented as mean ± standard deviation, number (%), or median (interquartile range). NRS, nutritional risk screening; NRS 2002 change = NRS at discharge − NRS at referral; ICU, intensive care unit; ^a^ patient group excluding death, ^b^ good compliance group (*n* = 128), bad compliance group (*n* = 127).

**Table 3:** Risk factors of high-risk malnutrition at discharge based on NRS 2002 ^a,b^.

	B	S.E.	OR	OR 95% CI		*p*-Value
Constant	−2.93	2.09	0.05			0.161
Bad compliance	1.54	0.50	4.68	1.74	12.63	0.002
NRS 2002 at referral	0.44	0.19	1.55	1.05	2.29	0.024
Ventilator care status	0.29	0.63	1.34	0.38	4.71	0.641
CCI	0.09	0.10	1.09	0.90	1.33	0.363
Length of ICU stay	0.03	0.01	1.03	0.99	1.07	0.056
Age	0.02	0.01	1.02	0.98	1.05	0.268
Length of hospital stay	0.01	0.01	1.01	0.98	1.03	0.331
BMI at referral	−0.18	0.06	0.83	0.73	0.93	0.003
Wound status	−0.60	0.64	0.54	0.15	1.92	0.346

CCI, Charlson Comorbidity Index; ICU, intensive care unit; BMI, body mass index; NRS, nutritional risk screening; B, regression coefficient; OR, odds ratio; SE, standard error. ^a^ Logistic regression included variables significant in the univariate analysis. ^b^ Patient group, excluding deaths (*n* = 204). CI: confidence interval.

## Data Availability

Data supporting the findings of this study are available from the corresponding author [S.-H.P.] upon reasonable request.

## Supplementary Materials

The following supporting information can be downloaded at: https://www.mdpi.com/article/10.3390/jcm13123422/s1, Table S1: Comparison of consensus recommendations for daily micronutrient administration.
