# Supplementary material for: Assessing the Impact of Nutritional Support Teams on Clinical Outcomes: Compliance and Feasibility of Micronutrient Supplementation
Source: J Clin Med. 2024 Jun 11;13(12):3422. doi: 10.3390/jcm13123422 (PMC11204540; doi:10.3390/jcm13123422)
Supplement: Supplementary file 1 [file jcm-13-03422-s001.zip › jcm-2975210-supplementary.pdf]

Table S1. Comparison of consensus recommendations for daily micronutrient administration

Vitamin(IV)	Consensus (upper reference)	Multivitamin Supply	Fulfill rate%
Vitamin A (IU)	3500	3300	94
Vitamin C (mg)	200	100	50
Vitamin D (IU)	200	200	100
Vitamin E (mg)	10	10	100
Vitamin K	Individual assessment	0	0
Vitamin (B1) Thiamin (mg)	6	3.8	63
Vitamin (B2) Riboflavin (mg)	5	3.6	72
Vitamin (B6) Pyridoxine	6	4.8	80
Vitamin (B3) Niacin (mg)	47	40	85
Biotin (mcg)	60	60	100
Vitamin(B9) (mcg)	600	400	67
Vitamin (B12) (mcg)	6	5	83
Vitamin (B5) (mg)	17	15	88
Trace elements(IV)	Consensus (upper ref)	Trace element Supply	Fulfill rate%
Zn (mg)	6.5	0.9	14
Cu (mg)	0.6	0.18	30
Cr (mcg)	15	1.8	12
Fe (mg) (Recommending)	1.2	0	0
Mn (mcg)	100	90	90
Se (mcg)	100	100	100
Mo (mcg)	No recommendation	0	0
I (mcg) (Recommending)	150	0	0
